# Cylindroma of the breast: a case report and review of the literature

**DOI:** 10.1186/1746-1596-4-30

**Published:** 2009-09-02

**Authors:** Amr Mahmoud, David H Hill, Martin J O'Sullivan, Michael W Bennett

**Affiliations:** 1 BreastCheck, National Cancer Screening Service/Mercy University Hospital, Cork, Ireland; 2 BreastCheck, National Cancer Screening Service/South Infirmary Victoria University Hospital, Cork, Ireland

## Abstract

Cylindroma of the breast is a very rare lesion which is morphological and immunophenotypically identical to benign dermal cylindroma. We report a breast cylindroma in a previously healthy 62 year old female detected through a national breast screening program. The patient had no significant family or past medical history, and specifically no history of breast or skin diseases. The tumor consisted of well circumscribed islands of epithelial cells surrounded by a dense membrane material, and focally containing hyaline globules. At low power the islands of tumour cells formed a "jig-saw" pattern, which is typical of cylindroma, but was present within normal breast parenchyma and no had direct connection with the overlying skin. Two distinct cell populations, smaller peripheral basaloid cells and larger central cells with vesicular chromatin, were highlighted by immunohistochemistry for p63 and cytokeratin-7 respectively. Immunohistochemistry for ER, PR, and Her2/neu was negative in tumour cells. We discuss the nine previously reported cases and the distinction of breast cylindroma from adenoid cystic carcinoma, the main differential diagnosis.

## Background

Cylindroma of the breast is a rare benign entity. To date only nine cases have been published to our knowledge. Although the appearance of cylindroma of the breast is identical to that of its dermal counterpart these lesions arise within the breast parenchyma. It is usually present as a solitary lesion, or may be associated with other lesions. We present a case detected by a national mammographic screening program. We review the radiology, histology, clinical course and discuss the previous cases in the literature. We discuss its differentiation from the main differential diagnosis, solid variant of adenoid cystic carcinoma.

## Case presentation

**Figure 1:**
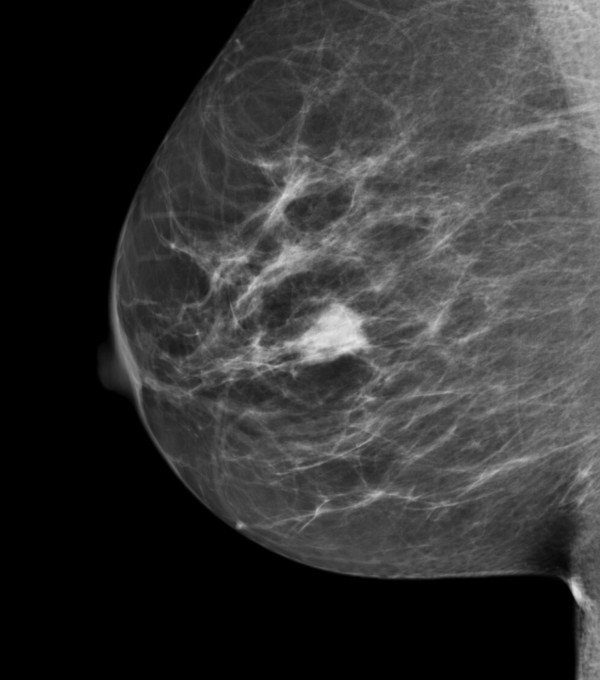
**Screening mammogram image, mediolateral oblique view**. A circumscribed mass is seen in the 3 o'clock position of the right breast.

**Figure 2:**
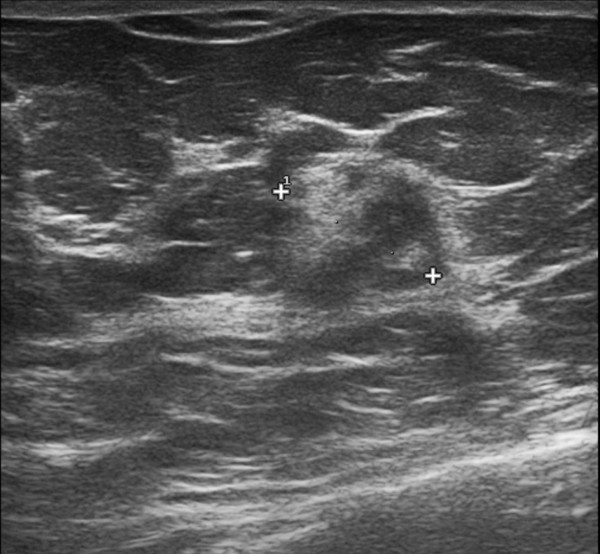
**Ultrasound screening image**. A corresponding heterogeneous mass shows increased echogenicity.

A 62 year old woman was invited to present for breast screening by the National Breast Cancer Screening Service (BreastCheck). She had neither significant medical nor family history. A 1.6 cm circumscribed reasonably defined mass was detected on screening mammography in the 3 o'clock position of her right breast (Figure 1). Ultrasound imaging showed a corresponding heterogeneous mass containing areas of notably increased echogenicity (Figure 2). On physical examination the lesion was not palpable. There were no skin or nipple changes, and no axillary or supraclavicular adenopathy. The lesion was considered suspicious but probably benign, and thus accorded a radiological grading (NHS breast screening system) score of M3 and U3 on mammographic and ultrasonic imaging respectively. An ultrasound guided core needle biopsy was performed. The patient underwent a right breast excision. The excision specimen weighted 13 gm, and measured 3.8 × 3.2 × 1.7 cm. Specimen radiography revealed a central density.

**Figure 3:**
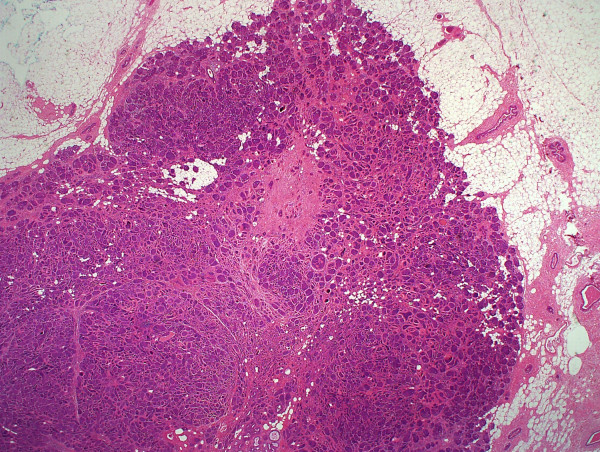
**Cylindroma at Low power view**. The characteristic "jig-saw" appearance of the cylindroma present in normal breast tissue.

**Figure 4:**
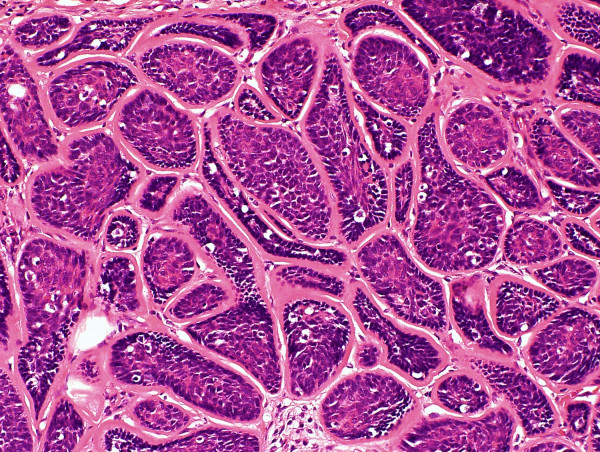
**High power magnification of the cylindroma**. Nests of basaloid cells are surrounded by a dense hyaline basement membrane.

**Figure 5:**
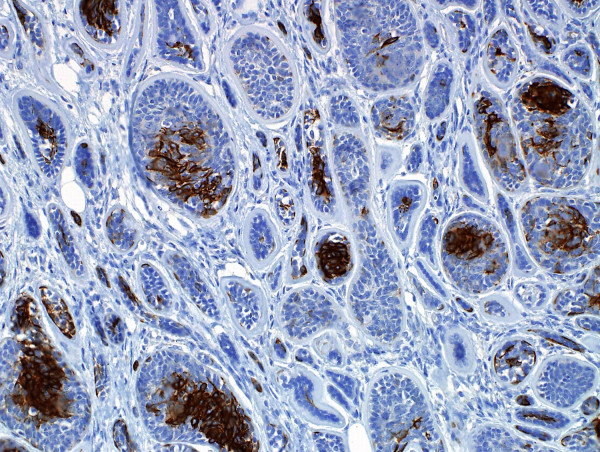
**Immunohistochemistry for cytokeratin-7 stains**. The central larger cells are highlighted.

**Figure 6:**
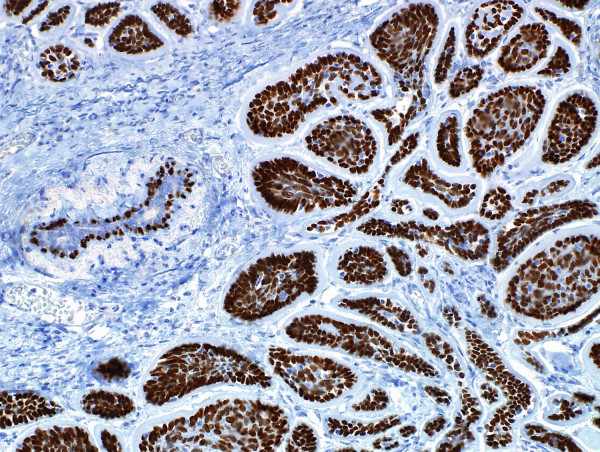
**Immunohistochemistry for P63**. Diffuse intense nuclear positivity, exaggerated at the periphery and diminished at the centre of the cell. nests.

On serially sectioning the specimen a firm tumour measuring 1.6 cm in maximum dimension was identified. Representative sections were submitted. An unencapsulated relatively circumscribed tumor was identified on microscopy. The tumor was composed of tightly arranged nests of cells with a characteristic "jig-saw" or "mosaic" appearance at low power (Figure 3). Surrounding each nest were prominent dense bands of eosinophilic basement membrane material (Figure 4). The nests of cells were composed predominantly of small, basaloid cells with scant cytoplasm and hyperchromatic nuclei. At the center of the nests the cells had increased eosinophilic cytoplasm and vesicular nuclei. Focally globules of hyaline material were also present within the nests. Occasional ducts, and a single breast lobule, indistinguishable from those of the normal breast, were also present within the tumor. There were no mitotic figures, necrosis or nuclear pleomorphism. Apoptotic bodies were focally present and were most prominent at the center of the nests. The surrounding uninvolved breast tissue was unremarkable. The histopathological diagnosis of benign eccrine cylindroma, which had been rendered on the core biopsy, was confirmed. To delineate the immunohistochemical features of the tumor staining was performed. The central basaloid cells were positive for cytokeratin-7 (CK7) while cytokeratin-20 stain was negative throughout (Figure 5). The tumor cells showed intense nuclear positivity for p63 which was exaggerated at the periphery of the cell nests and was diminished at the center (Figure 6). Prominent dendritic langerhans cell were noted on S100 stain. Scattered ducts within the tumour were positive for EMA, and also very focally positive for polyclonal CEA, consistent with eccrine differentiation. Immunhistochemstry for ER, PR and Her2/neu was negative in tumor cells, but ER and PR stained the scattered breast ducts and lobule which were present within the tumor. The patient's post-operative course was uneventful and routine follow-up was arranged. The tumor had not recurred at six months post-diagnosis.

## Case Discussion

Firstly, discribed in 2001. Cylindroma of the breast is such a rare entity, for only 9 cases were published in the literature [1-4]. Cylindromas presented in five cases as symptomatic masses, in one case as a lesion at mammography, and in three cases as incidental findings in excisions performed for invasive carcinoma; ductal type in two cases and lobular type in another. Six cases arose in the left breast and three in the right breast. In three cases the cylindromas were retroareolar, and in five cases they arose adjacent to lactiferous ducts. Our case had neither of these features. Two cases were associated with familial cylindromatosis. All cases were treated with excision except one patient with an infiltrative ductal carcinoma who had a mastectomy. Two patients who also had an infiltrating carcinoma underwent axillary dissection which showed metastatic breast carcinoma to lymph nodes but no involvement by cylindroma. None of eight patients had recurrence with follow-up ranging from six months to five years. One patient was lost to follow up. All lesions were identical in morphological appearance to dermal cylindromas. They consist of nests and trabeculae of cells that interlock in a "jigsaw" or "mosaic" pattern. The nests are composed of peripheral basaloid cells, central larger cells with oval vesicular nuclei, occasional clusters of sebaceous cells and squamous cells, and scattered reactive langerhans cells. A markedly thickened basement hyaline membrane, which is immunoreactive to collagen IV, and PAS surrounds the epithelial nests. Hyaline globules, composed of basement membrane material, are focally present within the epithelial nests. Focal eccrine ducts are present and may contain secretions. Diffuse positivity for p63 in the basaloid cells and CK7-positivity in the larger central cells support the morphological impression of two divergent types of cellular differentiation; myoepithelial and ductal. The extent of positive nuclear staining for p63 supports predominant myoepithelial differentiation in these tumors. Breast cylindromas occur entirely within the breast parenchyma with no connection to the overlying skin.

Dermal cylindromas are relatively common benign skin adnexal neoplasms. They usually occur in the sixth decade of life, with a male preponderance of 1:9. They present most commonly on the head, neck, or scalp as slowly growing, pink to purple, solitary or multiple, smooth surfaced nodules, which can rarely grow and coalesce to produce the characteristic turban-like mass (turban tumor). Most are asymptomatic but they can be painful when associated with spiradenoma. Although morphologically identical one study suggested that dermal cylindromas as a group may have more langerhans cells and eccrine ducts than breast cylindromas [3]. Cylindromas have also been reported at other extracutaneous sites including salivary gland, bronchus, lung and kidney. Brooke-Spiegler syndrome or familial cylindromatosis is a rare familial condition, which is inherited in an autosomal dominant fashion, with variable expression and penetrance. The syndrome is associated with the occurrence, generally from childhood or adolescence, of multiple dermal cylindromas. The lesions are widely distributed not only on the head and neck, but also the trunk and extremities, and are occasionally associated with other dermal adnexal tumours such as spiradenoma [5].

In two of the nine published cases of breast cylindroma the patients had Brooke-Spiegler syndrome. The CYLD gene, which is responsible for this syndrome, has been localized to chromosome 16q12-q13, and probably represents a tumour suppressor gene [6]. Malignant transformation of dermal cylindromas is very rare but cases have been reported in the setting of Brooke-Spiegler syndrome. Malignant cylindromas often present with ulceration of the skin and display large epithelial cells with marked pleomorphism, numerous mitotic figures and obvious invasion, but usually there is a transition from benign to malignant [7].

A variety of neoplasms of eccrine and apocrine sweat glands have been reported in the breast. Their occurrence at this site is not surprising as the breast is an ectodermal derivative of the same cell lineage that gives rise to the cutaneous apocrine glands. In fact, many authors regard the breast as a modified sweat gland. The most common adnexal tumors occurring in the breast are mixed adnexal tumour and syringoma, which follow a benign clinical course in most cases. However cases of recurrent spiradenoma [8] and a carcinomasarcomatous eccrine spiradenoma [9] have been reported.

Cylindroma of the breast has been linked with the solid type of adenoid cystic carcinoma (ACC), basaloid variant in the literature. Morphologically, both tumours have a nodular and trabecular appearance, they also share basaloid cells, myoepithelial cells. Moreover, eccrine ducts structure and hyaline globules of basement membrane have been recognised in both tumours. Solid-type ACC can display moderate to marked nuclear atypia, invasive tumor borders, and brisk mitotic activity [10]. However, Cylindroma of the breast does not exhibit nuclear atypia or mitoses. ACC may be associated with mucin production, a feature which is not present in cylindroma. Recently, it has been reported that mammary ACC expresses c-kit in the luminal component [11]. However, in our case, the tumour nests didn't express any type of positivity for c-kit except for few mast cells present within the intervening stroma. While the two entities share a basal membrane that surrounds the cell nest, immunohistochemical stains for the collagen IV material reveals a thin, discontinuous band of membrane surrounding the nodules of ACC [12], in contrast to a thickened, continuous band of basement membrane that surrounds the epithelial nests of breast cylindromas. Although mammary ACC has an excellent prognosis, yet there have been reported cases with local recurrence and distant metastases [13]. The basaloid, solid-variant ACC runs an aggressive clinical course; it has been reported to metastasize to axillary lymph nodes [14]. On the other hand, breast cylindroma characteristically runs a completely benign course. All previous cases reported in the literature have been treated with wide local excision, have not recurred and have not metastasized to lymph nodes or to distant organs. Furthermore, mastectomy or excisional biopsy with adjuvant radiotherapy has been recommended as treatment of solid-variant of mammary ACC [10].

## Conclusion

Breast cylindroma is a rare benign tumor. It should be clearly distinguished from adenoid cystic carcinoma as the implications for prognosis and managment of these lesions are obviously different.

## Consent

Written informed consent was obtained from the patient for publication of this case report and any accompanying images. A copy of the written consent is available for review by the Editor-in-Chief of this journal.

## Competing interests

The authors declare that they have no competing interests.

## Authors' contributions

AAM was involved in the literature search, histopathology evaluation, preparing the materials, and drafting the manuscript. DHH provided the screening radiography details and figures. MJOS supplied the relevant clinical details MWB outlined the general concept, interpreted the histopathology, and revised the manuscript. All authors have read and approved the present manuscript.
